# Knowledge, Attitudes, and Practices towards Silver Diamine Fluoride among Dentists in Vietnam

**DOI:** 10.3390/dj12060169

**Published:** 2024-06-05

**Authors:** Hollis Haotian Chai, Quang Khai Dao, Trong Hung Hoang, Sherry Shiqian Gao, Edward Chin Man Lo, Chun Hung Chu

**Affiliations:** 1 Faculty of Dentistry, The University of Hong Kong, Hong Kong 999077, China; htchai89@connect.hku.hk (H.H.C.); sherrysgao@xmu.edu.cn (S.S.G.); hrdplcm@hku.hk (E.C.M.L.); 2 Faculty of Odonto-Stomatology, University of Medicine and Pharmacy at Ho Chi Minh City, Ho Chi Minh City 72714, Vietnam; daoquangkhai@ump.edu.vn; 3 Department of Stomatology, School of Medicine, Xiamen University, Xiamen 361005, China

**Keywords:** silver diamine fluoride, caries, children, mixed-methods research

## Abstract

Silver diamine fluoride (SDF) is a topical solution used for managing dental caries. The mixed-methods study consists of a quantitative study (questionnaire survey) and a qualitative study (in-depth interview) to explore the knowledge, attitudes, and practices towards SDF among dentists in Vietnam. A descriptive approach and a thematic approach were performed to analyze data, respectively. The questionnaire survey invited 436 licensed dentists registered for the national annual dental meeting and 226 dentists participated (response rate: 52%). Among them, 174 (77%, 174/226) dentists knew SDF, and 69 (40%, 69/174) dentists used SDF for caries management. Most of them considered SDF therapy as non-invasive (84%, 146/174) and simple (80%, 140/174). However, most of them expressed concern that SDF could discolor the tooth (74%, 128/174). Their most preferred teeth for SDF therapy were primary posterior teeth (92%, 160/174). The in-depth interview consulted 16 dentists to reach data saturation. They learned about SDF from outside curriculum resources as an effective anti-caries agent. They understood the advantages (simple, non-invasive, timesaving) and disadvantages (tooth discoloring, ammonia odor) of SDF. They used SDF to arrest caries in uncooperative children in the clinic and people living in rural areas in outreach services. Most dentists in Vietnam are supportive of SDF therapy, and they know its advantages and disadvantages for caries management. The results addressed the aim of the study to investigate Vietnamese dentists’ perspectives towards SDF.

## 1. Introduction

The burden of dental caries represents a significant public health concern globally, affecting individuals across all age groups. This can lead to considerable treatment costs and disparities in access to dental care services [1]. The management of dental caries has changed in recent years from being primarily invasive to being preventive and involving minimally invasive procedures. This change is primarily driven by the growing understanding of the importance of preserving tooth structure and reducing patient discomfort [2]. One such intervention that has received increased attention from researchers and practitioners is silver diamine fluoride (SDF). 

The shift towards preventive methods, including SDF therapy, has significant implications for improving patient outcomes, reducing treatment costs, and promoting overall oral health. SDF is a colorless solution containing silver and fluoride ions. It was developed in Japan in the 1960s by Professor Yamaga and Professor Nishino from Osaka University [3]. SDF has been considered to be a safe, effective, and cost-efficient method for arresting dental caries [4]. In 2014, the United States Food and Drug Administration (FDA) approved the use of SDF for treating dentin hypersensitivity, and in 2017, it granted breakthrough therapy designation for the approval of SDF as a drug to treat severe early childhood caries [5]. Clinical evidence also supports that SDF can have a significant and substantial benefit in arresting and preventing caries across the age spectrum [6,7]. 

In 2021, the World Health Organization (WHO) included SDF in the WHO List of Essential Medicines for both adults and children [8]. Despite the potential advantages of SDF, its adoption and integration into dental practices vary across different countries and regions [9]. Dentists may have concerns regarding the permanent discoloration of carious teeth caused by SDF treatment. The specific concerns include the aesthetic impact of the darkened lesions, which may lead to the potential refusal of SDF therapy. This discoloration issue can be a barrier to the widespread adoption of SDF therapy, as patients may prioritize the appearance of their teeth over the benefits of the treatment [10].

Vietnam is a Southeast Asian country with a population of over 96 million. It has been experiencing rapid socio-economic growth in recent years. Despite this progress, oral health remains a public health issue in the country. The national oral health survey in 2019 reported the caries prevalence of 5-year-old children was 86%. Their caries experience in terms of dmft was 6.2. More than 90% of the caries remained untreated. Fluoride usage in caries management is generally low in Vietnam, with only 4% of the population having access to optimally fluoridated water [11]. There is also a lack of widespread fluoride toothpaste usage [12]. 

In addition, access to dental care remains limited, particularly in rural areas where the dentist-to-patient ratio is significantly lower than in urban areas [13]. The study showed that urban subjects had less decay (about 70%) but more fillings than rural subjects in Vietnam, which also indicated that the rural region has poor access to dental treatment [14]. Vietnam’s high caries prevalence, coupled with inequity in access to dental care and low fluoride exposure, makes it an ideal country to explore SDF therapy for caries management and prevention.

Dentists’ utilization of silver diamine fluoride (SDF) varies across different regions, particularly in Southeast Asia, where dental education on SDF remains unstandardized, which can create challenges such as inconsistent knowledge among practitioners, limited training access, and varied patient acceptance. Additionally, unclear guidelines and protocols may cause confusion among dentists, hindering the widespread adoption of this effective treatment option [15]. Considering the identified research gap, this study addresses the limited exploration of dentists’ perspectives on SDF in Vietnam. 

The high caries experience and prevalence in Vietnam warrant the need for a pragmatic approach to caries prevention. The country presents an ideal setting to conduct a study, delving deeper into the understanding of SDF utilization among Vietnamese dentists. The objective of this study is to explore the knowledge, attitudes, and practices towards SDF therapy among dentists in Vietnam. Specifically, the research aims to address the following questions: (1) What level of knowledge do dentists possess regarding SDF therapy? (2) What are the prevailing attitudes among dentists towards SDF therapy, including the effectiveness and perceived pros and cons? (3) How do these knowledge and attitudes influence the adoption and implementation of SDF therapy in their practices? By investigating these research questions, the study seeks to identify potential barriers and facilitators to the widespread adoption of SDF therapy in Vietnam.

The findings from this study hold significance not only in enhancing our comprehension of SDF utilization in Vietnam but also in contributing to the global understanding of SDF application among dental professionals. Specifically, these results shed light on the dentists’ knowledge, attitudes, and practices related to SDF therapy, which can inform the development of targeted educational programs and guidelines.

Additionally, by identifying potential barriers and facilitators to SDF adoption, this study offers insights for policymakers and dental organizations worldwide to address similar challenges and promote the broader integration of SDF therapy into dental practice. In doing so, the study’s findings contribute to enhancing oral healthcare outcomes and supporting the global advancement of evidence-based dentistry.

## 2. Methods

**Figure 1:**
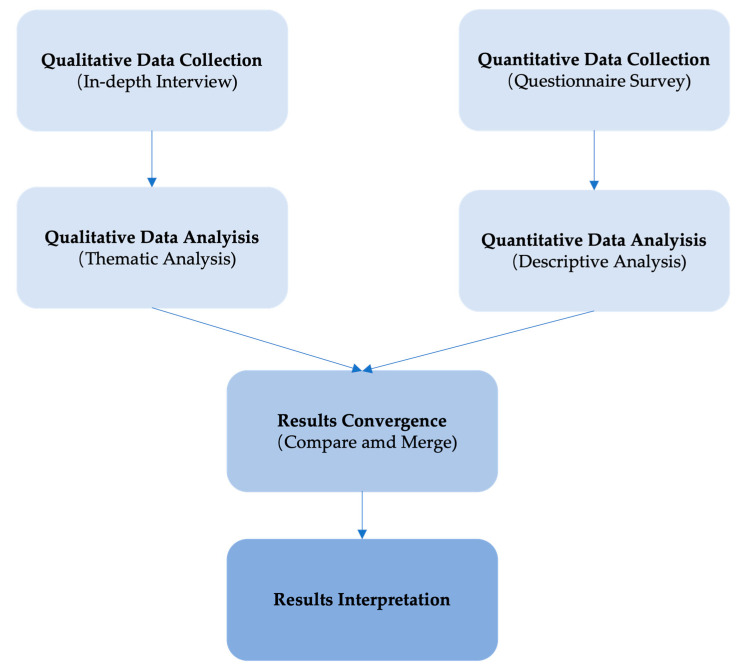
Concurrent Convergence Parallel Triangulation Design of a mixed-methods study.

This study employed a mixed-methods study design, which incorporated both qualitative study and quantitative study. The quantitative study was an online questionnaire survey, whereas the qualitative study was individual in-depth interviews. While an online questionnaire survey was used in the quantitative analysis, individual in-depth interviews were used in the qualitative investigation. This study used the Concurrent Convergence Parallel Triangulation Design (Figure 1), which enabled data collection within the same timeframe (concurrent), merging through convergence design, equal weighting (parallel), and the utilization of multiple methods to examine the same issues (triangulation) [16]. This methodology was adopted from a published study conducted in Japan [17]. The University of Medicine and Pharmacy at Ho Chi Minh City reviewed and offered ethics approval for this study (Approval No.578/HĐĐĐ-ĐHYD).

### 2.1. Quantitative Study

The quantitative study was an online questionnaire survey to gather demographic information of the participants, including their dental education, current practice, and position. It also assessed the dentists’ knowledge, practices, and attitudes towards SDF therapy.

The pilot study indicated that 70% of Vietnamese dentists were aware of SDF. The margin of error and confidence level were set as 5% and 90%, respectively. This resulted in a necessary sample size of 229. Considering an anticipated response rate of 60%, a minimum of 382 dentists had to be invited to participate in this questionnaire survey.

Table 1 summarizes the content of the questionnaire.

The questionnaire was adopted from our published study assessing the knowledge, attitudes, and practices towards SDF among dentists in Japan [12]. Two researchers who were fluent in both English and Vietnamese from the Department of Dental Public Health of the University of Medicine and Pharmacy in Ho Chi Minh City adapted the questionnaires to align with the cultural context of Vietnam. They translated the questionnaire into Vietnamese by two independent bilingual speakers. Backward translation was performed to ensure semantic equivalence by another two independent bilingual translators. They compared the back-translated English version to the original English version and made necessary further revisions based on the results of the semantic equivalence assessment and pilot test. After that, the questionnaire was set up in a web-based format. An invitation email containing a brief introduction to the study and a link to the online questionnaire was sent to all 436 licensed dentists registered in the dental annual meeting in Vietnam in 2023. A reminder email was dispatched to these dentists one month after the meeting. The two researchers collated the responses and extracted the collected data into Excel. They performed data cleaning and conducted descriptive analysis. Chi-squared (or Fisher exact) test was performed to compare the participating dentists’ perspectives on SDF therapy indications based on their different demographic characteristics. SPSS version 29.0 (IBM Corp., Chicago, IL, USA) was used for the statistical analysis.

### 2.2. Qualitative Study

The qualitative study employed individual in-depth interviews to collect the interviewees’ perspectives on SDF therapy. The researchers identified dentists who used SDF in the government, institutional, and private sectors. The first phase of this qualitative study invited nine dentists. They participated and formed the first cohort of interviewees. The researchers employed a snowball sampling method to recruit more dentists for interviews. In each interview, the last question posed was, “Could you please recommend any dentists who may have valuable insights on SDF therapy?” This allowed the interviewees to introduce more potential participants who are experienced in SDF therapy. Regular meetings were held by the researchers and investigators throughout the study to assess the data gathered and discuss the study’s progress. They continued recruiting interviewees until no more new information was collected (i.e., data saturation).

Two trained researchers (HHC and QKD) who were proficient in English and Vietnamese conducted the interview in either Vietnamese or English depending on the interviewee’s preference. The researchers facilitated the interviews according to the interview guide developed in our previous study [12]. Table 2 shows the contents of the interview guide.

The researchers took fieldnotes and audio-recorded the discussion. They transcribed the audio records verbatim and conducted a thematic data analysis. They familiarized themselves with the data by continuously reviewing interview transcripts and identifying relevant topics. They refined and sorted these topics to create a thematic framework, independently coded the transcripts, and generated a codebook for data coding through discussions and evaluations. The researchers regularly reviewed and refined codes using the codebook, making necessary revisions. Finally, they summarized the data according to the constructed themes and identified connections and associations between them.

Following the separate analyses of the collected quantitative and qualitative data, the findings were compared and merged through a narrative approach. During the interpretation and reporting stage, they adopted the contiguous approach to integration by presenting the findings in separate sections within a unified report. The fit of data integration was rigorously evaluated to check the coherence of the quantitative and qualitative findings.

## 3. Results

### 3.1. Demographic Information

#### 3.1.1. Quantitative Study—Demographic Information

The quantitative survey invited 436 dentists, and 226 dentists completed the questionnaire. The response rate was 52%. Table 3 shows the dentists’ demographic information. Most of them (74%, 168/226) practiced not more than 10 years.

#### 3.1.2. Qualitative Study—Demographic Information

Sixteen separate in-depth interviews were conducted by two researchers, and data saturation was attained. Eleven of them were in-person interviews, and the remaining five interviews were Zoom interviews. The average time for the interviews was 29 min and the time ranged from 17 min to 45 min.

### 3.2. Knowledge of SDF Therapy

#### 3.2.1. Quantitative Study—Knowledge of SDF Therapy

Of the 226 dentists who responded, 174 (77%, 174/226) indicated they were aware of SDF. Among those, 174 dentists familiar with SDF, 119 (68%, 119/174) reported having engaged in discussions about SDF with their colleagues, and 93 (53%, 93/174) had introduced SDF to patients.

#### 3.2.2. Qualitative Study—Knowledge of SDF Therapy

In general, the interviewed dentists knew, and most of them had basic knowledge regarding SDF. Their understanding was mainly centered around the composition of SDF and its effectiveness for caries control. However, there exists a notable polarization in the depth of knowledge of SDF therapy among Vietnamese dentists. Some experienced dentists have been using SDF for over six years. They actively engaged in discussing SDF with their students and colleagues. 

Conversely, some dentists who had merely heard of it lacked clinical experience in SDF therapy. Their knowledge was primarily acquired through external sources such as workshops, conferences, and introductions from friends rather than dental curricula in their dental schools.

*I have been using SDF for six to seven years. So, I share my experience with my colleagues.*

—Interviewee No. 4

*We just heard about it, but we haven’t practiced it.*

—Interviewee No. 7

*SDF is not part of the formal lecture curriculum at the university.*

—Interviewee No.13

### 3.3. Attitudes towards SDF Therapy

#### 3.3.1. Quantitative Study—Attitudes towards SDF Therapy

Table 4 illustrates the dentists’ attitudes on the effectiveness of SDF. Most dentists who knew SDF (81%, 141/174) concurred SDF to be effective for caries prevention but only 77 dentists (44%, 77/174) acknowledged the desensitizing effect of SDF.

Table 5 shows the dentists’ perspectives on the advantages and disadvantages of SDF therapy. Most dentists who knew SDF concurred that SDF therapy was non-invasive (84%, 146/174), but only 65 dentists (37%, 65/174) considered SDF therapy to be inexpensive. In addition, most of the dentists considered that SDF therapy resulted in an unaesthetic appearance (74%, 128/174), but only 67 dentists (39%, 67/174) considered the unpleasant taste a disadvantage of SDF use.

#### 3.3.2. Qualitative Study—Attitudes towards SDF Therapy

The interviewed dentists acknowledged the effectiveness of SDF in arresting dental caries and reducing dentin hypersensitivity, citing growing research that strengthens the evidence base. They specifically mentioned its success in hardening caries lesions. However, they expressed concerns about patient cooperation during treatment and communication challenges in explaining SDF therapy. While they praised SDF’s short-term effectiveness in caries management, they also conveyed uncertainty regarding its long-term efficacy due to patients’ inconsistent oral hygiene maintenance.

*I didn’t verify its long-term effectiveness due to the lack of patient cooperation.*

—Interviewee No. 2

*I find SDF effective in halting caries progression and arresting its growth.*

—Interviewee No. 13

*I find SDF very effective, especially for small caries lesions… Initially, there weren’t enough research results, but now, with more evidence-based research, I trust the technique more. We now have more evidence to support its use.*

—Interviewee No. 15

The interviewed dentists recognized the advantages of SDF, including its simplicity, non-invasive nature, and time-saving application. They also knew that SDF therapy was quick and painless and could alleviate patients’ anxiety and fear associated with dental treatment. Furthermore, they emphasized that patients tended to accept SDF well, primarily due to its non-invasive nature.

*It does not require drilling the teeth, which makes it less frightening for children.*

—Interviewee No. 1

*I find SDF easy to use, it doesn’t cause pain, and it increases patient cooperation.*

—Interviewee No. 2

*It can be applied to many teeth at once, making it a convenient and time-saving option.*

—Interviewee No. 3

The most concerning disadvantage of SDF mentioned by the interviewed dentists was the black-discoloring property of SDF. Patients could complain about the unaesthetic appearance, which not only came from the staining of carious teeth but also from inadvertent staining of the skin of the patient’s face and operator’s hand. Furthermore, some dentists were concerned about the pungent smell and unpleasant taste of SDF. Some of them also worried about SDF, which might cause gingival irritation.

*If not applied carefully, it can irritate the gingiva.*

—Interviewee No. 1

*Another concern is the taste. I tasted it. It’s awful. It’s bitter.*

—Interviewee No. 4

*One disadvantage is that it causes black staining on the treated area and has a sour taste.*

—Interviewee No. 14

### 3.4. Practice of SDF Therapy

#### 3.4.1. Quantitative Study—Practice of SDF Therapy

Sixty-nine dentists have provided SDF therapy, and 42 dentists were using SDF on their patients. Table 6 shows dentists’ perspective on indications of SDF therapy. 

Among the 174 dentists who know about SDF, 79 dentists (45%, 79/174) considered that it should always be used to arrest caries in primary molars, but 68 dentists (39%, 68/174) considered that SDF should never be used to arrest root caries. Additionally, a significant proportion of dentists were inclined to opt for SDF to prevent caries in primary teeth (84%,147/174). No statistically significant outcome (*p* > 0.05) was observed in the Chi-squared (or Fisher exact) test of dentists’ perspective on SDF therapy indications (Table 6), based on the demographic characteristics (No. of years of practice; Primary employment; Have postgraduate degree or diploma) of participating dentists (Table 1).

#### 3.4.2. Qualitative Study—Practice of SDF Therapy

The interviewed dentists regarded the use of SDF mainly for caries control in uncooperative children, in particular for those with rampant caries or high caries risk. The dentists suggested using SDF for arresting caries in primary anterior teeth, occlusal surfaces of primary posterior teeth, and caries that were not advanced or close to the dental pulp. Some dentists affirmed SDF therapy for older adults. A few dentists mentioned SDF therapy to treat dentin hypersensitivity. One dentist mentioned using SDF in adults by applying potassium iodide (KI) after SDF application to reduce black staining. The dentist also suggested using SDF to arrest caries of the tooth before endodontic therapy and prevent secondary caries and enhance the long-term success of restorations.

*For uncooperative patients, I apply SDF on front teeth and occlusal surfaces of back teeth.*

—Interviewee No. 2

*For adults, I worry about the black staining. So, I combine SDF with KI…… I use SDF on adult patients requiring endodontic therapy. I use SDF to harden decay and protect the tooth after filling.*

—Interviewee No. 4

*SDF can also be effective to treat difficult-to-access caries lesions in elderly patients because conventional restoration might not be feasible.*

—Interviewee No. 15

The interviewed dentists stated that SDF was not suitable for deep caries or patients with irreversible pulpitis, as they were concerned about potential allergic reactions. They mentioned applying SDF before restoring the tooth with glass ionomer cement (GIC). They also used SDF for caries control before they employed restorative treatment in subsequent visits. One dentist mentioned using SDF with laser irradiation to arrest caries lesion cavities and to desensitize teeth with hypersensitivity.

*As for contraindications, SDF is not suitable for patients with deep caries or those with symptoms of irreversible pulpitis.*

—Interviewee No. 3

*Sometimes, I combine SDF with laser treatment for patients with sensitivity issues.*

—Interviewee No. 4

*If the cavity is not too deep and not causing much discomfort, I use SDF and cover it with GIC.*

—Interviewee No. 9

Although the interviewed dentists knew the merits of SDF therapy, some of them did not consider SDF therapy as the primary choice for caries management. They had concerns about the black staining by SDF resulting in patient dissatisfaction and even rejection of subsequent dental care. As a result, dentists often opted for conventional restorations as the primary choice for cooperative and adult patients. Furthermore, the dentists mentioned that the use of SDF was more common in private clinics rather than university clinics or government hospitals due to a lack of adoption in hospital management systems and limited knowledge about SDF among general practitioners. Consequently, dentists faced barriers in incorporating SDF into their practice within institutional settings.

*For patients with cooperative behaviors, I consider other treatment options like restorations.*

—Interviewee No. 3

*I‘ve asked the parents, and they don’t want to use it because it makes the teeth too black…… In our clinic, we can use it. However, in our hospital and department, we have to follow certain regulations.*

—Interviewee No. 5

Dentists in Vietnam expressed the challenges they faced in applying SDF therapy and its patient feedback. One of the major challenges is the difficulty in communication with parents, which affects children’s treatment with SDF. They emphasized the importance of effective communication and education of the patients and caregivers, focusing on maintaining good oral hygiene. Dentists stressed the need to educate parents about the therapy, helping them understand and accept the treatment, and ensuring regular follow-up visits. In terms of clinical application, dentists faced challenges such as isolating the cavity. Although dentists usually recommend applying SDF therapy twice a year, it sometimes ends up being a one-off application due to the loss of patient follow-up.

*The decision to use SDF depends on the individual patient’s cooperativeness, usually only in the first treatment.*

—Interviewee No. 1

*When deciding to use SDF, I spend a lot of time discussing the pros and cons with the patient and their parents so they understand the black staining and potential effectiveness.*

—Interviewee No. 9

*The first problem is consulting parents about the black staining of the teeth. Another issue is the difficulty of isolating the teeth properly while applying SDF, as children tend to produce a lot of saliva.*

—Interviewee No. 11

The interviewed dentists highlighted SDF therapy in community settings, particularly in underserved areas where dental care is not available and people have low awareness of oral health. They emphasized its potential to improve the oral health of the people in rural areas, where people’s aesthetic concerns might not be the priority. Some dentists shared their experience of using SDF outreach dental care in kindergarten or primary schools. Furthermore, SDF therapy could be beneficial for special needs populations, such as individuals with autism or disabilities. They found variations in the SDF application protocols in community settings, such as isolation procedure, SDF application time, and post-treatment instruction such as the need and the time for refraining from drinking or eating or the need to rinse or spit out the excess SDF solution.

*I think it’s possible to use SDF for other people. For example, if they cannot go to a dental office, we can apply it for them at home, if possible.*

—Interviewee No. 8

*I believe SDF has great potential in community-based projects, especially in areas where dental care is limited. I have participated in community projects where SDF was used to provide dental care to children in need, and the results were quite encouraging.*

—Interviewee No. 9

*SDF can also be beneficial for special needs patients, as well as those with autism or disabilities who may have difficulty maintaining their oral health.*

—Interviewee No. 16

## 4. Discussion

This study represents the first exploration of dentists’ perspectives on SDF therapy in Vietnam. The SDF, developed in Japan, has been utilized by clinicians there for over 50 years since the 1960s [18]. The study found that in terms of knowledge, only 77% of respondents had heard of SDF, compared to 100% in the previous study conducted in Japan [17]. Moreover, most interviewed Vietnamese dentists gained their understanding of SDF from external sources rather than formal dental curricula. This highlights the need for better integration of SDF education within the dental education system in Vietnam, taking into account the country’s unique social and developmental context.

The attitudes towards SDF’s preventive potential exhibited discrepancies between quantitative and qualitative results. The survey indicated that most participating dentists perceived SDF as effective in preventing dental caries, and most of them were willing to consider SDF for caries prevention in primary teeth. However, the interviewed dentists did not mention SDF’s preventive potential in their clinical use. A similar situation was observed regarding the desensitizing effect of SDF, where positive attitudes did not translate into active practice, leading to inconsistencies between attitudes and practices. This disparity might be attributed to insufficient teaching on certain SDF applications, such as caries prevention and treatment of tooth hypersensitivity, within dental schools in Vietnam. The previous study investigating the SDF teaching landscape in dental schools across Southeast Asia further corroborates this finding [15]. Also, a study in Saudi Arabia evaluating general practitioners’ SDF clinical experience, knowledge, professional behavior, and attitudes found that only 63% of the participating dentists had heard of SDF, and there was a lack of knowledge even among the dentists who knew SDF, which also align with this study and indicates the importance of SDF education for the dental professionals [19].

The observed discrepancies between quantitative and qualitative results regarding attitudes towards SDF’s preventive potential might be influenced by social norms and the socio-economic context of Vietnam. The social norms have led to a coexistence of traditional and Western medicine in Vietnam [20]. Cultural beliefs could impact dentists’ decisions to incorporate SDF into their practice, as they may be more inclined to recommend treatments that align with their patients’ beliefs and values. Limited healthcare resources, concerns over patient acceptance, and insufficient public awareness of SDF’s benefits may also contribute to these disparities [21]. By examining these underlying factors, strategies can be developed to promote SDF adoption in dental practice, ultimately improving patient outcomes in Vietnam. These strategies may include continuing education, public awareness campaigns, and guideline development.

Prior research has demonstrated that dentists who possess greater knowledge about SDF tend to exhibit more positive attitudes towards its use and are more likely to employ it in their practice [22]. This finding was also observed in the present study. Another national survey in the US assessed U.S. pediatric dentists’ SDF educational experiences, knowledge, attitudes, and professional behavior [23]. The survey found that despite the relatively low level of SDF education in respondents’ predoctoral and graduate programs, their knowledge about SDF use was quite high. This highlights the importance of professional development education on SDF use. Comparing our findings with these previous studies provides additional insights into factors influencing SDF therapy adoption in Vietnam. Furthermore, despite most dentists utilizing SDF in pediatric populations and community settings, there remains a lack of standardized application procedures. Factors such as application time, the necessity of mouth rinsing, and differences between SDF solution and gel formulations are still under investigation in various clinical trials. This highlights the need for further research and education to establish evidence-based guidelines for SDF application, thereby ensuring its optimal use in dental practice.

The social context in Vietnam reveals that many parents prioritize filling cavities over using SDF due to concerns about black staining. This may be a reflection of the country’s cultural values and expectations regarding oral health and appearance. Additionally, primary teeth are often considered unimportant, leading to limited treatment. This attitude may be influenced by a lack of public awareness and understanding of the importance of primary teeth, which could be addressed through targeted education and outreach efforts. Comparatively, more than 70% of the Vietnamese population is not connected to public water supplies, limiting the expansion of fluoridation coverage [11]. In this context, promoting SDF in Vietnam may be particularly beneficial for underserved populations, special needs population, and those living in rural areas with limited access to dental care.

To advance the adoption of SDF in Vietnam, several strategies can be considered. Firstly, incorporating SDF education into dental curricula through lectures and practical sessions [15]. This will help ensure that future dental professionals have a comprehensive understanding of SDF and its potential benefits. Secondly, aligning the promotion of SDF with the WHO Essential Medicines List [8] and the Action Plan for Oral Health in South-East Asia 2022–2030 [24] can help ensure its successful implementation in the region. This alignment would demonstrate international support for SDF and provide a strong foundation for its adoption in Vietnam. Thirdly, fostering collaboration with universities and international partners can aid in expanding the scope of research and promoting SDF usage. Sharing experiences and new research findings with global partners can facilitate the development of effective strategies for SDF implementation in Vietnam. Additionally, engaging in global discussions on generating guidelines for SDF use can help ensure that standardized protocols are established, further enhancing the safe and effective utilization of SDF in dental practice [9].

Several challenges need to be addressed when promoting SDF in Vietnam. First, communication with patients is essential, as dental professionals need to effectively convey the benefits of SDF and address potential concerns, such as black staining [25]. Developing culturally appropriate educational materials and communication strategies can help alleviate these concerns and improve patient acceptance. Second, concerns about black staining and the relatively new status of the material may lead to hesitancy among both dental professionals and patients [13]. Addressing this challenge requires targeted education efforts to increase the awareness and understanding of SDF and its advantages. Third, there may be logistical challenges related to the procurement and distribution of SDF in Vietnam, particularly in remote or underserved areas. Ensuring the accessibility and affordability of SDF will be crucial to its successful adoption across the country [26].

As further collaboration with universities and other countries continues, the scope of research on SDF and its implementation in various settings is expected to expand. This will provide valuable insights into the effectiveness of SDF therapy in different patient populations and contexts, including underserved communities, special needs patients, and those with limited access to dental care. Additionally, the ongoing development and dissemination of new research findings will contribute to the evidence base supporting SDF, further promoting its adoption in Vietnam and beyond [4]. As more countries recognize the benefits of SDF and include it in their oral health policies and programs, it is anticipated that the global acceptance and utilization of SDF will continue to grow [9].

The strength of the study is that it employed a mixed-methods study design. Mixed-methods research combines the strengths of both qualitative and quantitative research methodologies [27]. Mixed-method research offers a comprehensive approach to address complex research questions, including those found in the field of dentistry. Qualitative research is suitable for exploring people’s behavior, attitudes, beliefs, and personality characteristics [28]. It offers a fresh perspective on investigating issues related to dental knowledge and dental clinical practice [29]. At the same time, quantitative research contributes to the generalizability and reliability of findings by providing statistical data and measurable outcomes. Qualitative and quantitative research can be executed concurrently or sequentially, with equal or differential prominence given to each aspect [30]. The integrative approach of mixed-methods study allows researchers to explore various dimensions of a research question, cross-validate findings, and generate more nuanced, contextualized insights [31]. This study also used a concurrent triangulation design. Data collection was convergent parallel [32]. Ultimately, this methodological pluralism equips researchers with a versatile toolkit to tackle multifaceted research problems and contribute to a richer understanding of the subject matter [33]. The quantitative findings and qualitative findings mutually enhanced each other, providing stronger inferences than using either approach on its own [34].

Mixed-methods research can provide valuable insights but has its own study design limitations. The time-consuming and resource-consuming nature of data collection and analysis for both qualitative and quantitative components may have constrained the study’s scope and depth. Additionally, the complexity of integrating data from different sources could have limited the comprehensiveness of the findings. This can lead to higher costs and increased complexity in project management. A team of researchers with expertise in both quantitative and qualitative methodologies is necessary. Therefore, mixed-methods research is suitable only when the research questions warrant its application [35]. Despite these constraints, the mixed-methods approach employed in our study was instrumental in generating a more nuanced understanding of SDF adoption and usage in the Vietnamese context.

The study has its limitations. Firstly, despite sending two reminders to increase participation, only 52% of the invited dentists completed the questionnaire survey. This group may not entirely represent the views of all invited dentists, potentially creating bias. However, there is no established benchmark for an acceptable response rate, and web surveys typically yield lower response rates [36]. Secondly, the generalizability may be considered as a limitation due to the response rate. However, robust mixed-methods research (incorporating a thorough integration of qualitative and quantitative phases and data) is often considered more suitable for producing valid generalizations, as it merges qualitative and quantitative frameworks, methodologies, and methods for various objectives [37]. Moreover, generalizability in qualitative research is often assessed differently, with a greater focus on a contextualized comprehension of human experiences and the transferability to comparable contexts [38]. Therefore, despite the potential limitation in generalizability, this mixed-methods study still provides valuable insights into the knowledge, attitudes, and practices of Vietnamese dentists concerning SDF therapy.

## 5. Conclusions

In conclusion, most dentists in Vietnam are familiar with SDF therapy, and they are supportive of its use, especially for children and outreach services. Although most dentists understand its advantages and disadvantages, their adoption and use of SDF therapy remain limited. Addressing the challenges and limitations identified in this study will be beneficial for promoting the successful implementation of SDF therapy across various settings.

## Figures and Tables

**Table 1:** Questions of the online questionnaire survey.

*Domain 1. Demographic data*
What is your Dental Practice (Government or Institution/Private/Both)
Dental Education (Basic/Advanced) No. of years of Dental Practice
Primary Location of Practice (City/Rural Area)
*Domain 2. Knowledge*
Have you ever heard of SDF therapy?
Have you ever introduced SDF therapy to other dentists?
Have you ever introduced SDF therapy to your patients?
*Domain 3. Attitudes*
Which of the following would you consider as advantages of SDF therapy? Please give your answer to EACH of the following five items. 1 Simple/2 Short application time/3 Non-invasive/4 Inexpensive/5 Painless
Which of the following would you consider as disadvantages of SDF therapy? Please give your answer to EACH of the following seven items. 1 Unaesthetic /2 Unpleasant taste/3 Stains items/4 Toxicity/5 High fluoride content/ 6 High silver content
Can SDF therapy prevent dental caries?
Can SDF therapy arrest dental caries?
Can SDF therapy desensitize dentin hypersensitivity?
Can SDF solution detect dental caries?
*Domain 4. Practices*
Have you ever used SDF to treat your patient in the clinic?
Are you using SDF to treat your patient in clinic?
How many patients did you treat by using SDF during last month?
Will you use SDF to manage caries in the following situations? Please give your answer to EACH of the following seven situations 1 Prevent caries in primary teeth/2 Prevent caries in permanent teeth/ 3 Arrest primary anterior caries/4 Arrest primary posterior caries/ 5 Arrest permanent anterior caries/6 Arrest permanent posterior caries/7 Arrest root caries
Will you deliver SDF therapy to the following populations? Please give your answer to EACH of the following seven populations 1 Preschool children/2 Primary school students/ 3 Secondary school students/4 Aged 18-34/ 5 Aged 35-64/6 Non-institutionalized adults aged 65 or above/7 Functionally dependent adults aged 65 or above Will you deliver SDF therapy the following people with special needs? Please give your answer to EACH of the following two populations 1 People with mental disorders/2 People with physical disabilities/

**Table 2:** Contents of the interview guide.

Key Question(s)	Follow-up Question(s)
*Domain 1. Background information*	
1.1 When did you get your basic dental training?	How long is the basic dental training?
1.2 Which school did you study for your basic dental training?	What is your highest education level attained?
1.3 What is your current position?	In which department do you work?
*Domain 2. Knowledge*	
2.1 When do you know about SDF?	How do you learn about SDF?
2.2 Where do you learn about SDF?	In what curriculum/program did you learn about SDF? Through what media did you learn about SDF?
2.3 What information of SDF have you shared in your professional or teaching activities?	In what curriculum have you taught SDF therapy? What basic knowledge of SDF have you delivered? What clinical use(s) of SDF have you discussed?
*Domain 3. Attitude*	
3.1 How effective is SDF in caries management?	How effective is SDF in arresting childhood caries? How effective is SDF in arresting adult caries? How effective is SDF in arresting root caries?
3.2 What are the advantages and disadvantages of SDF therapy?	What are the merits of using SDF? What are the indications of using SDF? What are the limitations of using SDF? What are the contra-indications of using SDF?
3.3 What are the challenges or barriers of SDF use in clinical care?	What are the clinical-related barriers of using SDF? What are the barriers in other aspects of using SDF?
*Domain 4. Practices*	
4.1 How do you use SDF in your dental practice?	What kinds of patients will you use SDF? How often do you apply SDF to your patients?

**Table 3:** Demographic information of the participating dentists (*n* = 226).

Items	Categories	No. of Dentists (%)
Years of practice	10 years or less	168 (74%)
	Over 10 years	58 (26%)
Main employment	Private practice	119 (53%)
	Government or institution	60 (26%)
	Both	47 (21%)
Higher dental training	Yes	106 (47%)
	No	120 (53%)
Have heard of SDF	Yes	174 (77%)
	No	52 (23%)

**Table 4:** Dentists’ attitudes on the effectiveness of SDF therapy (*n* = 174).

Effectiveness of SDF (Mean, SD)	Categories	No. of Dentists (%)
Prevent caries (2.8, 0.46)	Agree (3)	141 (81%)
	Neutral (2)	29 (17%)
	Disagree (1)	4 (2%)
Arrest caries (2.3, 0.71)	Agree	82 (47%)
	Neutral	68 (39%)
	Disagree	24 (14%)
Desensitize dentin hypersensitivity (2.4, 0.58)	Agree	77 (44%)
	Neutral	89 (51%)
	Disagree	8 (5%)
Detect dental caries (2.0, 0.67)	Agree	39 (22%)
	Neutral	96 (56%)
	Disagree	39 (22%)

**Table 5:** Dentists’ attitudes on the advantages and disadvantages of SDF therapy (*n* = 174).

Items (Mean, SD)	Categories	No. of Dentists (%)
***Advantages***		
Simple (2.8, 0.42)	Agree (3)	140 (80%)
	Neutral (2)	33 (19%)
	Disagree (1)	1 (1%)
Short application time (2.8, 0.44)	Agree	133 (76%)
	Neutral	40 (23%)
	Disagree	1 (1%)
Non-invasive (2.8, 0.36)	Agree	146 (84%)
	Neutral	28 (16%)
	Disagree	0 (0%)
Inexpensive (2.3, 0.64)	Agree	65 (37%)
	Neutral	90 (52%)
	Disagree	19 (11%)
Painless (2.8, 0.47)	Agree	134 (77%)
	Neutral	37 (21%)
	Disagree	3 (2%)
***Disadvantages***		
Unaesthetic (2.7, 0.6)	Agree	128 (74%)
	Neutral	34 (19%)
	Disagree	12 (7%)
Contamination of color (2.7, 0.58)	Agree	123 (71%)
	Neutral	42 (24%)
	Disagree	9 (5%)
Unpleasant taste (2.3, 0.56)	Agree	67 (39%)
	Neutral	99 (57%)
	Disagree	8 (4%)
Toxic (1.6, 0.54)	Agree	5 (3%)
	Neutral	101 (58%)
	Disagree	68 (39%)
Harmful due to high silver content (1.7, 0.56)	Agree	9 (5%)
	Neutral	107 (62%)
	Disagree	58 (33%)
Harmful due to high fluoride content (1.6, 0.60)	Agree	11 (6%)
	Neutral	90 (52%)
	Disagree	73 (42%)

**Table 6:** Dentists’ perspective on SDF therapy indications (*n* = 174).

Use of SDF Therapy (Mean, SD)	Frequency of Use	No. of Dentists (%)
To prevent caries in primary teeth (2.1, 0.65)	Always (3)	49 (28%)
	Sometimes (2)	98 (56%)
	Never (1)	27 (16%)
To prevent caries in permanent teeth (1.7, 0.68)	Always	21 (12%)
	Sometimes	74 (43%)
	Never	79 (45%)
To arrest primary anterior caries (2.1, 0.66)	Always	43 (25%)
	Sometimes	98 (56%)
	Never	33 (19%)
To arrest primary posterior caries (2.4, 0.63)	Always	79 (45%)
	Sometimes	81 (47%)
	Never	14 (8%)
To arrest permanent anterior caries (1.5, 0.69)	Always	19 (11%)
	Sometimes	41 (24%)
	Never	114 (65%)
To arrest permanent posterior caries (1.7, 0.73)	Always	29 (17%)
	Sometimes	67 (38%)
	Never	78 (45%)
To arrest root caries (1.8, 0.70)	Always	26 (15%)
	Sometimes	80 (46%)
	Never	68 (39%)

## Data Availability

The datasets generated and/or analyzed during the current study are available from the corresponding author upon reasonable request.
